# Supplementary material for: Toxic Metals in Particulate Matter and Health Risks in an E-Waste Dismantling Park and Its Surrounding Areas: Analysis of Three PM Size Groups
Source: Int J Environ Res Public Health. 2022 Nov 21;19(22):15383. doi: 10.3390/ijerph192215383 (PMC9691227; doi:10.3390/ijerph192215383)
Supplement: Supplementary file 1 [file ijerph-19-15383-s001.zip › ijerph-1968145-supplementary.pdf]

Supplementary Materials

Toxic metals in particulate matter and health risks in an e-waste dismantling park and its surrounding areas: Analysis of three PM size groups

Yingjun Wu [1,2], Guiying Li [1,2,*] and Taicheng An [1,2]

1 Guangdong-Hong Kong-Macao Joint Laboratory for Contaminants Exposure and Health, Guangdong Key Laboratory of Environmental Catalysis and Health Risk Control, Institute of Environmental Health and Pollution Control, Guangdong University of Technology, Guangzhou 510006, China

2 Guangzhou Key Laboratory of Environmental Catalysis and Pollution Control, Key Laboratory of City Cluster Environmental Safety and Green Development of the Ministry of Education, School of Environmental Science and Engineering, Guangdong University of Technology, Guangzhou 510006, China

* Correspondence: ligy1999@gdut.edu.cn; Tel.: +86-15323343835

There are 13 pages including 5 figures and 7 tables.

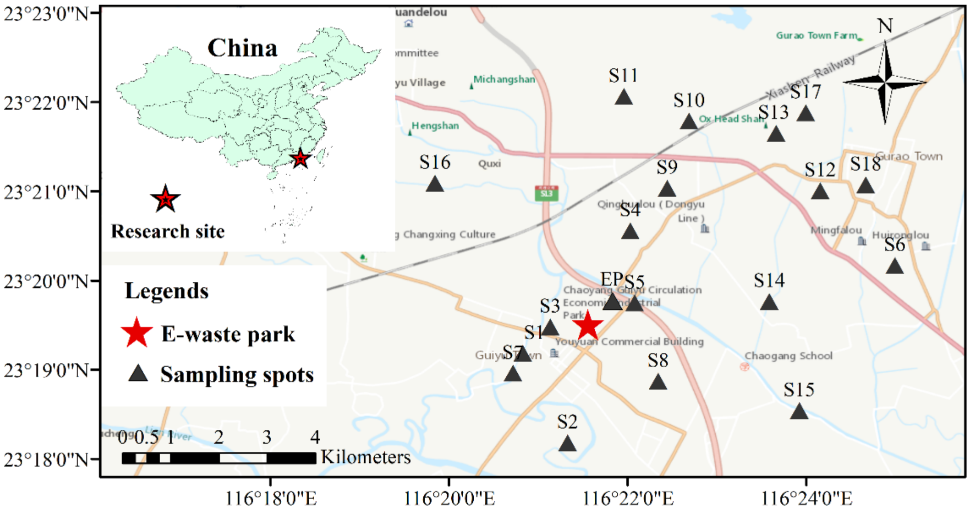

Figure S1. Research site and sampling spots in e-waste area

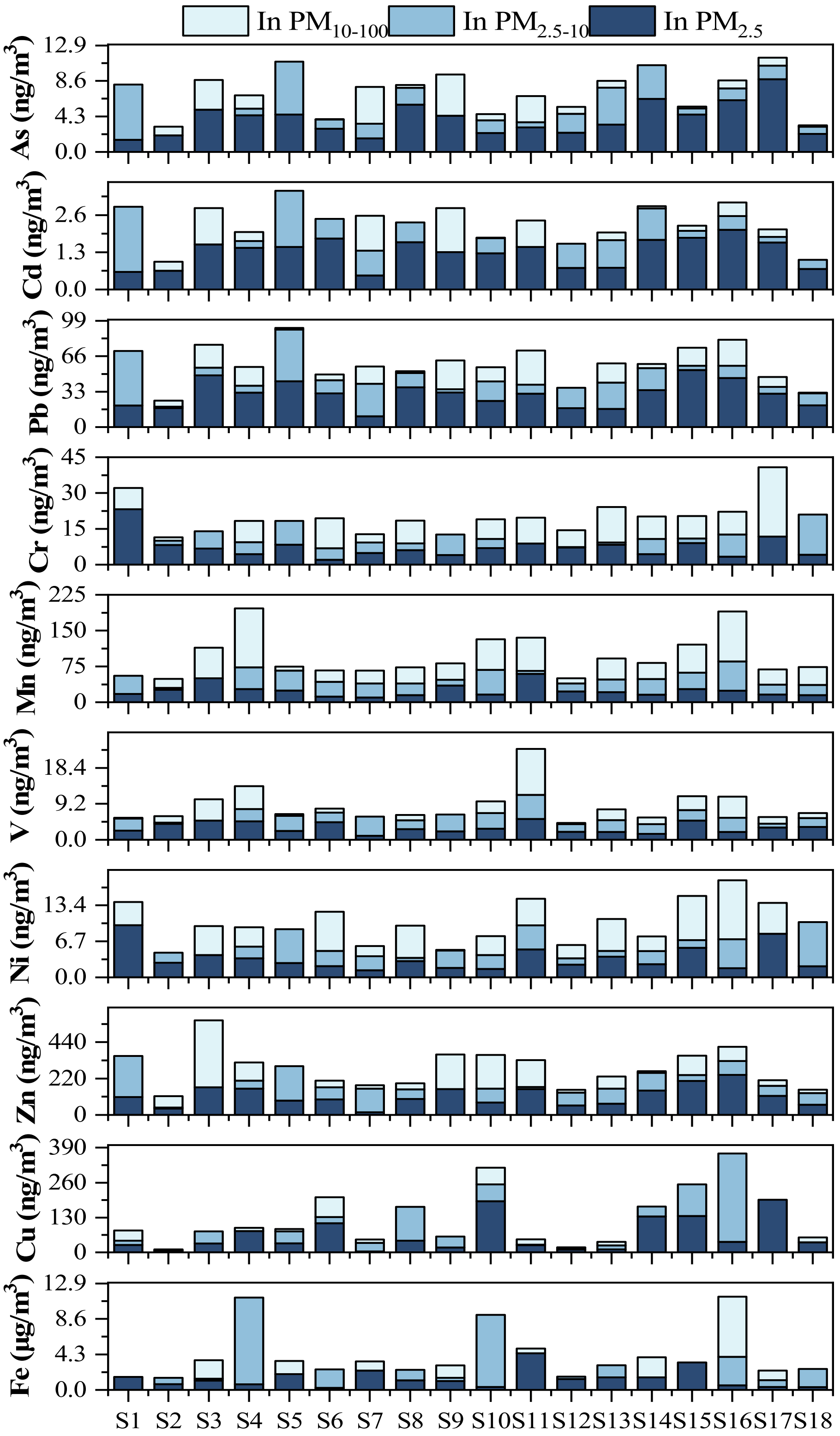

Figure S2. Metal concentration in each site of the e-waste surrounding residential area

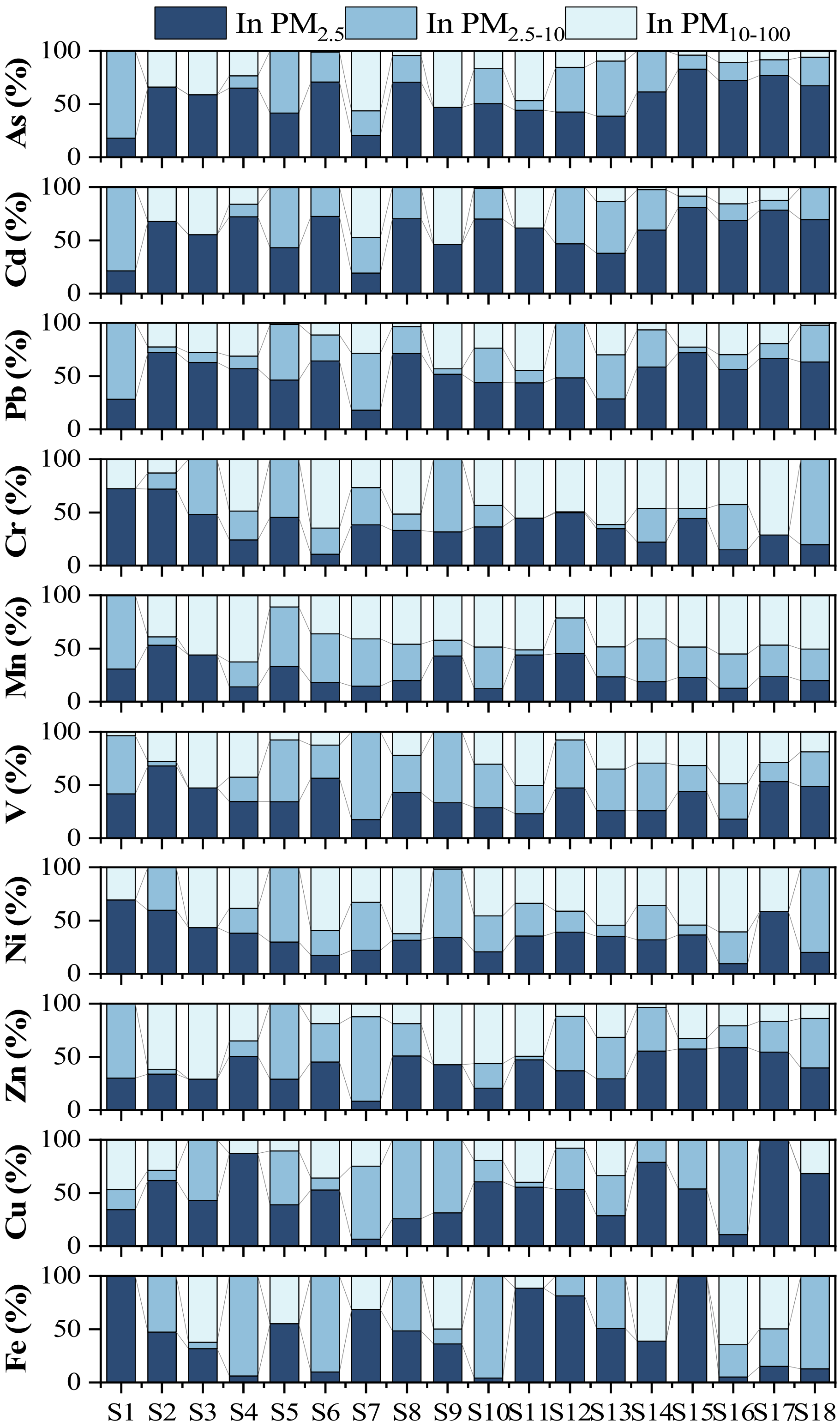

Figure S3. Size distribution of metals in each site in the residential areas of e-waste area

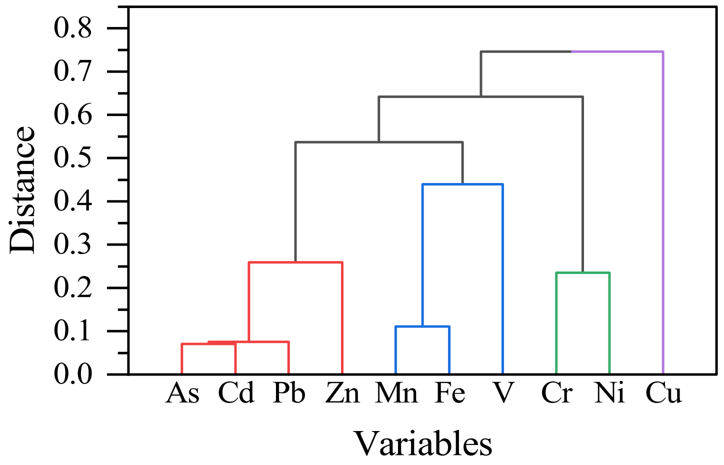

Figure S4. The cluster analysis of metals in the surrounding residential area; the same color represents the primary cluster

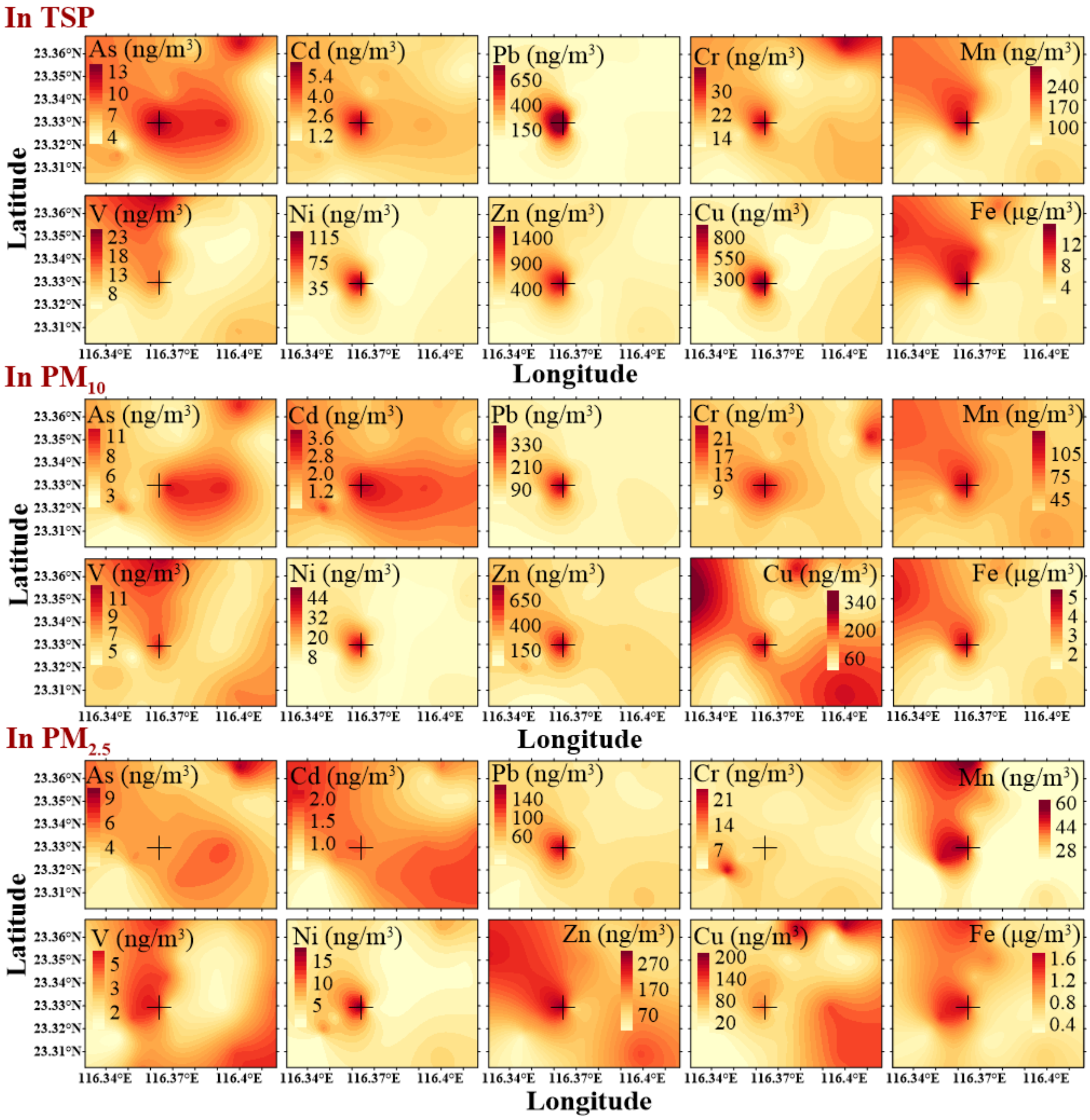

Figure S5. Spatial distribution of metal in TSP, PM10 and PM2.5 in e-waste dismantling park (the location of cross) and surrounding residential areas; wind direction is mainly northeast.

Table S1. The longitude and latitude of sampling spots

Sampling date	Sampling spot	Longitude (N)	Latitude (E)	Temperature (°C)	Weather
2017.11.2	EP	116°21'06"	23°19'02"	34.4	Sunny
2017.11.2	S1	116°22'06"	23°19'38"		
2017.11.3	S2	116°21'36"	23°18'02"	34.9	Sunny
2017.11.3	S3	116°21'24"	23°19'20"		
2017.11.4	S4	116°22'18"	23°20'25"	32.5	Cloudy with scattered light rain
2017.11.4	S5	116°22'22"	23°19'27"		
2017.11.5	S6	116°25'16"	23°20'01"	32.8	Sunny
2017.11.5	S7	116°20'59"	23°18'49"		
2017.11.6	S8	116°22'37"	23°18'44"	32.2	Cloudy
2017.11.6	S9	116°22'43"	23°20'53"		
2017.11.7	S10	116°22'58"	23°21'39"	32.4	Cloudy to light rain
2017.11.7	S11	116°22'14"	23°21'55"		
2017.11.8	S12	116°24'26"	23°20'52"	33.6	Cloudy to sunny
2017.11.8	S13	116°23'56"	23°21'30"		
2017.11.9	S14	116°23'52"	23°19'37"	32.7	Sunny
2017.11.9	S15	116°24'12"	23°18'24"		
2017.11.10	S16	116°20'07"	23°20'57"	36.0	Sunny
2017.11.10	S17	116°24'17"	23°21'44"		
2017.11.11	S18	116°24'56"	23°20'56"	31.0	Cloudy

Table S2. Digestion program of microwave-assisted acid digestion method

Procedure	Climbing time/min	Duration /min	Temperature / ºC	Power/ W
1	15:00	5:00	170	1600
2	08:00	8:00	180	1600
3	08:00	15:00	200	1600
Total		56:00		

Table S3. The selected metals IUR and RfC values for health risk assessment

	As	Cr	Ni	Cd	V	Mn	Pb
IUR (mg/m^3)	4.3E-03	8.40E-02	2.6E-04	1.8E-03	-	-	8.0E-05
RfC (ug/m^3)$^{-1}$	1.5E-05	1.00E-04	9.0E-05	1.0E-05	1.0E-04	5.0E-05	-

Obtained from: https://www.epa.gov/risk/regional-screening-levels-rsls-generic-tables

Table S4. The Shapiro-Wilk normality test of metals concentration in size fraction in the surrounding residential area in e-waste area

		DF	Statistic	p-value	Decision at level (5%)
	$PM_{2.5}$	18	0.92887	0.18553	Can't reject normality
As	$PM_{2.5-10}$	18	0.88691	0.03418	Reject normality
	PM_{10-100}	18	0.87081	0.01835	Reject normality
	$PM_{2.5}$	18	0.904	0.06749	Can't reject normality
Cd	$PM_{2.5-10}$	18	0.93149	0.20624	Can't reject normality
	PM_{10-100}	18	0.88539	0.0322	Reject normality
	$PM_{2.5}$	18	0.96126	0.62614	Can't reject normality
Pb	$PM_{2.5-10}$	18	0.83408	0.0048	Reject normality
	PM_{10-100}	18	0.92663	0.16939	Can't reject normality
	$PM_{2.5}$	18	0.75589	3.86E-04	Reject normality
Cr	$PM_{2.5-10}$	18	0.92648	0.16831	Can't reject normality
	PM_{10-100}	18	0.88222	0.02846	Reject normality
	$PM_{2.5}$	18	0.82779	0.00386	Reject normality
Mn	$PM_{2.5-10}$	18	0.99212	0.99978	Can't reject normality
	PM_{10-100}	18	0.92946	0.18997	Can't reject normality
	$PM_{2.5}$	18	0.92499	0.15845	Can't reject normality
V	$PM_{2.5-10}$	18	0.9534	0.48079	Can't reject normality
	PM_{10-100}	18	0.84913	0.00821	Reject normality
	$PM_{2.5}$	18	0.81595	0.00257	Reject normality
Ni	$PM_{2.5-10}$	18	0.94031	0.2934	Can't reject normality
	PM_{10-100}	18	0.97651	0.90711	Can't reject normality
	$PM_{2.5}$	18	0.97013	0.80014	Can't reject normality
Zn	$PM_{2.5-10}$	18	0.94091	0.30035	Can't reject normality
	PM_{10-100}	18	0.89624	0.04944	Reject normality
	$PM_{2.5}$	18	0.81561	0.00255	Reject normality
Cu	$PM_{2.5-10}$	18	0.76532	5.12E-04	Reject normality
	PM_{10-100}	18	0.76956	5.82E-04	Reject normality
	$PM_{2.5}$	18	0.83224	0.0045	Reject normality
Fe	$PM_{2.5-10}$	18	0.88584	0.03277	Reject normality
	PM_{10-100}	18	0.71373	1.16E-04	Reject normality

Table S5. The recovery of metals in the blank quartz fiber filters with spiked 50 μg/L metals

Element	1#	2#	3#	Average concentrations	Standard deviation	Average recovery
Unit				μg/L		%
V	45.22	43.10	42.39	43.57	1.47	87.14
Cr	44.32	42.34	39.38	42.01	2.49	84.02
Mn	42.93	42.12	38.67	41.24	2.26	82.47
Ni	45.99	43.21	41.90	43.70	2.09	87.39
Cu	45.35	42.54	41.47	43.12	2.00	86.24
Zn	42.42	44.77	35.17	40.78	5.00	81.57
As	47.03	46.25	46.10	46.46	0.50	92.91
Cd	48.73	47.67	47.66	48.02	0.61	96.04
Pb	46.15	45.17	45.17	45.49	0.57	90.99

Table S6. The Spearman correlation of metals in e-waste dismantling park

	As	Cd	Pb	Cr	Mn	V	Ni	Zn	Cu
Cd	0.500								
Pb	0.500	1.000**							
Cr	-0.500	0.500	0.500						
Mn	0.500	1.000**	1.000**	0.500					
V	1.000**	0.500	0.500	-0.500	0.500				
Ni	0.500	1.000**	1.000**	0.500	1.000**	0.500			
Zn	0.500	1.000**	1.000**	0.500	1.000**	0.500	1.000**		
Cu	0.500	1.000**	1.000**	0.500	1.000**	0.500	1.000**	1.000**	
Fe	0.500	1.000**	1.000**	0.500	1.000**	0.500	1.000**	1.000**	1.000**

**: The correlation is significant at the 0.01 level (two-tailed);

Table S7. The Spearman correlation coefficients of metals in the residential area surrounding the e-waste dismantling park

	As	Cd	Pb	Cr	Mn	V	Ni	Zn	Cu
Cd	0.917**								
Pb	0.674**	0.668**							
Cr	-0.184	-0.164	-0.046						
Mn	-0.181	-0.286*	0.211	0.256					
V	0.048	0.057	0.392**	0.054	0.416**				
Ni	-0.188	-0.082	-0.008	0.860**	0.288*	0.239			
Zn	0.595**	0.589**	0.725**	0.089	0.363**	0.405**	0.206		
Cu	0.202	0.201	0.314*	0.137	0.169	0.223	0.145	0.362*	
Fe	-0.226	-0.265	-0.015	0.265	0.448**	0.129	0.264	0.010	-0.044

**: The correlation is significant at the 0.01 level (two-tailed);
*: The correlation is significant at the 0.05 level (two-tailed).
